# Is There a Relationship between Salivary Cortisol and Temporomandibular Disorder: A Systematic Review

**DOI:** 10.3390/diagnostics14131435

**Published:** 2024-07-05

**Authors:** Lujain AlSahman, Hamad AlBagieh, Roba AlSahman

**Affiliations:** 1 Oral Medicine and Diagnostic Sciences Department, College of Dentistry, King Saud University Riyadh, Riyadh 57448, Saudi Arabia; hamadnb@hotmail.com; 2 Faculty of Dentistry, Royal College of Surgeons, D02YN77 Dublin, Ireland; robaalsahman@gmail.com

**Keywords:** salivary cortisol, temporomandibular disorders (TMD), diagnostic tool, biomarkers

## Abstract

Background: This systematic review examines and evaluates the relationship between salivary cortisol levels and temporomandibular disorder (TMD) in young adult patients. Method: Six databases—PubMed, Scopus, Web of Science, Google Scholar, ProQuest, and Cochrane Library—were utilized to screen eligible studies. A systematic search was performed based on PECO questions and eligibility criteria. The research question for this review was “Do salivary cortisol levels correlate with TMD in individuals aged 18–40?” The risk of bias for quality assessment was determined by the Cochrane tool. PRISMA guidelines were followed while performing this review. Result: A total of fourteen studies were included in this review. Of these, eleven were observational studies (four cross-sectional and seven case–control), and three were randomized control trials. Eleven of the included studies presented a low to moderate risk in the qualitative synthesis. The total sample size of the included studies was 751 participants. The included studies suggest higher salivary cortisol levels in TMD patients than in healthy individuals. Conclusions: The findings of this review indicate higher salivary cortisol levels in adult patients with TMD than in healthy controls. Thus, supportive psychological treatment and clinical modalities should be provided to patients with TMD. Moreover, higher-quality studies with low heterogeneity are required to support this finding.

## 1. Introduction

The temporomandibular joint (TMJ) and its associated structures are pivotal in guiding mandibular movement and distributing stresses produced by everyday tasks like speaking, chewing, and swallowing [1]. Temporomandibular disorders (TMDs) are pathologic conditions affecting the TMJ, masticatory muscles, and surrounding tissues [2]. TMDs include abnormalities of the intra-articular disc’s position and/or structure and dysfunction of the associated musculature [3]. The symptoms of TMDs include a reduced range of movement of the mandible, painful joint sounds, masticatory muscle pain, TMJ pain, and restriction/deviation in mouth opening [4]. TMD is the most common cause of chronic orofacial pain due to non-dental origin [5], with the prevalence of TMDs being about 31% in adults and about 11% in children and adolescents [6]. The aetiology of TMD is considered to be multifactorial, including biomechanical (occlusal overload and parafunction), neuromuscular, biological (e.g., elevated levels of oestrogen hormones), and psychosocial (e.g., stress, anxiety, depression) factors [7]. Psychological factors, especially stress, are considered one of the main areas of TMD aetiology, but their role in the occurrence of TMD is inconclusive [8]. Stress is a normal physiological response in humans. However, it leads to pathological conditions when it exceeds the body’s adaptive capacity. It has also been found to strongly contribute to the formation and persistence of pain [9]. Additionally, pain and stress lead to parafunctional habits [9], like bruxism linked with signs and symptoms of TMD [8]. Mental stress is believed to stimulate the hypothalamic–pituitary–adrenal (HPA) axis, which triggers a series of reactions leading to increased cortisol secretion from the adrenal cortex [10]. As a product of the HPA axis, cortisol participates in anti-inflammatory and anti-stress activities and is considered a marker of stress and anxiety. Several studies have shown a correlation between HPA axis dysregulation and TMD [11,12]. Consequently, cortisol levels can be utilised to assess HPA axis activity [13] and evaluate TMD. Furthermore, cortisol level assessment can be developed as a diagnostic tool for TMD and to meet the specific treatment needs of TMD patients.

Cortisol levels can be measured using plasma, serum, urine, hair, and saliva specimens [14]. Considering its non-invasive mode of collection, saliva has become a good candidate for cortisol assessment. Moreover, salivary cortisol measurement indicates unbound active cortisol levels in contrast to bound plasma cortisol levels [15]. Previous studies have assessed salivary cortisol levels in TMD patients, with contradictory reports [16,17,18]. According to Kobayashi et al., no difference in salivary biomarkers was observed between children with and without TMD, although anxiety symptom scores were higher in children with TMD [18]. Contrary reports were provided by D’Avilla et al., who found significantly higher salivary cortisol values in their adult TMD group [17]. A recent study by Suprajith et al. concluded a significant impact of psychosocial stress on the etiopathogenesis of TMD [19]. Collaborating on these findings, Fritzen et al. stressed that the relationship between salivary cortisol and bruxism in adults and children could not be neglected [20]. Despite some existing studies, there is no conclusive evidence regarding the association of salivary cortisol levels in adult patients with TMD. Moreover, a significant increase in stress levels has been found in young adults compared to children and the elderly population. A literature search shows a systematic review by Lu et al. [21], but this review includes only case–control studies and a limited search period, from 2008 to 2020. Moreover, none of the existing studies focus on adult patients’ salivary cortisol concentrations. So, we aimed to search all the available data regarding salivary cortisol levels in adult TMD patients between 18–40 years old. Hence, this systematic review was conducted in order to determine whether a correlation exists between salivary cortisol levels and TMD in adult patients.

## 2. Materials and Methods

This systematic review evaluates the relationship between salivary cortisol levels and temporomandibular disorders (TMDs) in individuals aged 18–40.

### 2.1. Protocol and Registration

A preliminary literature search was run to identify the research problem, focus question, and eligibility criteria in January 2023. Following this, a protocol was prepared and registered with PROSPERO before the systematic review began (CRD42022378756).

### 2.2. Eligibility Criteria

The focus question was formulated using the PECO (population, exposure, comparison, and outcome) framework. Population (P) was young adults aged 18–40 with TMD and without any other oral pathology or disorder; exposure (E) was salivary cortisol levels measured in observational and randomized control trials; comparison (C) was healthy participants without TMD; and outcome (O) was changes in salivary cortisol levels in patients diagnosed with TMD. Consequently, the focus question was, “Do salivary cortisol levels correlate with temporomandibular disorders (TMDs) in individuals aged 18–40?”

The inclusion criteria for the studies were as follows: (1) studies including patients between 18–40 years of age, (2) studies including patients diagnosed with TMDs according to RDC/TMD or DC/TMD, (3) clinical studies (mainly case–control studies, randomized control trials, and observational studies), and (4) studies evaluating salivary cortisol/biomarkers for stress.

Studies were excluded using the following criteria: (1) case report/series, (2) conference papers, (3) in vitro studies, (4) editorials, (5) commentaries, (6) literature reviews, (7) animal studies, and (8) studies not in the English language.

### 2.3. Information Sources and Search Strategies

The PRISMA 2020 statement [22] was followed to report this systematic review. Two researchers performed the literature search on all major databases, such as PubMed, Scopus, Web of Science, Google Scholar, ProQuest, and Cochrane Library, to include all relevant studies related to TMDs and salivary cortisol levels. An experienced librarian was always available to assist with the literature search.

After developing the protocol, the initial search began in March 2023. The PECO criteria were utilized to run the search using keywords and MeSH terms related to temporomandibular disorders (“temporomandibular joint disorders” OR “temporomandibular joint dysfunction” OR “craniomandibular disorders” OR “TMJ osteoarthritis”) and salivary cortisol levels (Table 1), until December 2023 A manual search was also performed in the reference sections of already identified articles.

### 2.4. Study Selection

After identifying the articles from various databases, the titles and abstracts were screened independently by two reviewers to include all studies that satisfied the inclusion and exclusion criteria for this systematic review. This step removed duplicates using citation or reference manager (Endnote 21). Finally, two researchers retrieved and scrutinized full texts, and any disagreements were resolved by discussion.

### 2.5. Data Extraction and Data Items

Two reviewers extracted the following data from the full-text articles: author, year, country, study population, study design, sample size, test/control group size, age range, salivary cortisol levels in tests and controls, saliva collection techniques, the statistical significance of the results, and conclusion. The data retrieved were tabulated in Excel sheets. Both the reviewers confirmed the data obtained, and any disagreements were resolved by a third reviewer. The inter-examiner kappa coefficient between the examiners was measured and was above 0.9 for all the questions during data extraction.

### 2.6. Data Synthesis (Meta-Analysis)

The studies included in this systematic review are heterogenous in nature. Hence, no meta-analysis was planned. Overall, analysis of the studies included in this review was done by narrative summary of the included studies. The quality of the included studies was evaluated by deducing the risk of bias for randomized control trials, cross sectional studies, and case–control studies. Due to high heterogeneity among the included studies, the authors performed GRADE analysis to evaluate the certainty of the evidence of the studies included in this systematic review. The Grading of Recommendations Assessment, Development and Evaluation (GRADE) methodology for grading the certainty of evidence was utilized for the observational studies and RCTs [23].

### 2.7. Risk of Bias in Individual Studies

A panel comprising two researchers assessed the risk of bias among the included studies. The Cochrane Risk of Bias Tool II [24] was utilised for the risk of bias assessment of randomized control trials, and the Newcastle–Ottawa quality assessment scale was used for case–control studies [25]. The Newcastle–Ottawa quality assessment scale (adapted for cross-sectional studies) was applied for the risk of bias assessment of cross-sectional studies [26]. Scoring for the Newcastle–Ottawa scale (for cross-sectional and case–control studies) ranges from 0 to 10, with 0 meaning a high risk, 4 to 6 a medium risk, and 7 and above a low risk of bias. Scores for the Cochrane Risk of Bias Tool are measured as “low”, “high”, and “some concern”.

## 3. Results

**Figure 1:**
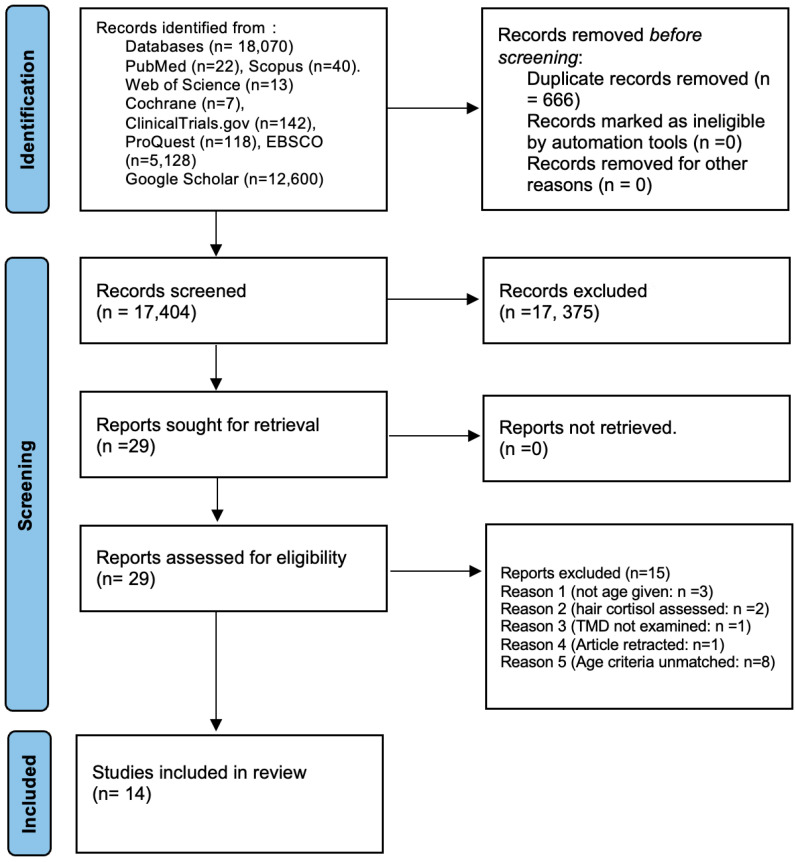
PRISMA flowchart.

The survey identified 18,070 studies on different databases, out of which 666 studies were duplicates. A total of 17,404 studies were directed to the reading of the title and abstract, out of which 17,375 were removed. A final 29 studies were selected for full-text reading, and 15 were removed for various reasons; the study selection process is explained in Figure 1.

Fourteen primary studies involving 751 participants (tests = 424 and controls = 327) met the criteria included in Table 2. The studies included in this review were published between 1997 and 2022.

### 3.1. Characteristics of Included Studies (Table 2)

Among the fourteen studies selected for data extraction were three randomized control trials [27,28,29], four cross-sectional studies [17,30,31,32], and seven case–control studies [16,33,34,35,36,37,38]. The included studies were from Brazil, Bosnia and Herzegovina, Thailand, Sweden, India, Canada, Iran, the USA, and China.

The study population in the test group consisted of young adult patients with TMD. All included studies—except one by Tosato et al.—had male and female participants, mostly university students. In the included studies, mainly female participants were affected by TMD, with higher stress and anxiety levels and elevated salivary cortisol [31]. As well as salivary cortisol levels, two studies by Rosar et al. studied bruxism and TMD [27,30]. Moreover, all three RCTs evaluated the effect of the treatment of TMD on salivary cortisol levels, and all of them reported a significant decrease in salivary cortisol levels after the treatment. The results of almost all included studies show that increasing salivary cortisol levels are seen in TMD patients with higher stress and anxiety levels. Goyal et al. [28] further verified that a significantly higher value of salivary cortisol was detected in TMD patients with depression than in TMD patients without depression, and treatment of TMD resulted in decreasing salivary cortisol levels. The Research Diagnostic Criteria for Temporomandibular Disorders (RDC/TMD) and clinical diagnosis were used to diagnose TMDs in almost all studies. The comparator group was mainly healthy controls without TMD. However, one study evaluated the salivary cortisol levels of TMD patients with normal occlusion and those with malocclusion [17]. A study by Magri et al. measured salivary cortisol levels with low-level laser therapy [29]. Goyal et al. measured salivary cortisol levels in cohorts of depressed and non-depressed TMD patients, as well as in a group of healthy controls [28]. Although the included studies were heterogenous, eleven of the included studies reported a higher salivary cortisol level in TMD patients than in controls.

### 3.2. Salivary Parameters of the Participants of the Included Studies (Table 3)

Seven primary studies used unstimulated saliva to assess cortisol (*n* = 381) [16,28,29,31,34,35,37], and the remaining five studies used stimulated saliva (*n* = 267) [17,27,30,36,38]. In seven studies, salivary samples were collected in the morning [27,29,30,31,32,34,37], while one study performed sampling both in the morning and evening [28]. However, in three studies, the timings of saliva sampling were not mentioned [17,33,36]. Six studies [16,28,33,34,36,37] collected saliva without extra stimulation, while five studies [17,27,29,30,35] collected saliva that was stimulated by chewing the swab and cotton roll. All the studies evaluated elevated cortisol levels with an ELISA kit, except for one study.

All the included studies utilized different measures for saliva collection. Quartana and colleagues collected salivary samples before and after 20 min of treatment [38]. While a stress test was performed by Jones et al., salivary samples were collected at three different intervals—at baseline, peak secretion (30 min), and 20 min after the rest [35].

Nine studies assessed salivary cortisol levels in adults with TMD compared to healthy controls. Seven of these were case–control study designs [16,33,34,35,36,37,38], and two were cross-sectional [31,32]. However, four of the included studies (three randomized control trials, and one cross-sectional study) evaluated salivary cortisol levels before and after treatment of TMD and controls. Ten of the included studies noted statistically significant differences between the salivary cortisol levels of both the groups. The statistical analyses reported in the included studies were in favour of the control group [17,27,28,29,30,31,32,33,34,37], showing higher cortisol levels in TMD patients. In contrast, four studies showed no statistical correlation between cortisol levels and TMD [16,35,36,38].

### 3.3. Risk of Bias Assessment (Table 4, Table 5 and Table 6)

The risk of bias assessment for the randomized controlled trials was performed by utilising the Cochrane Risk of Bias Tool-II [24]. One study was found to have a high risk [27], and two had a low risk of bias [28,29]. The high risk was due to inconsistency in measuring the exposure and differences in the sample allocation.

The Newcastle–Ottawa quality assessment scales for cross-sectional [26] and case–control studies [25] measured the risk of bias for corresponding studies. Among the seven case–control studies in the review, two had low risk [33,38], four had medium [16,34,35,36], and one had a high risk of bias [37]. The high- and medium-risk studies had higher heterogeneity in sample representativeness and exposure. Outcome measures were inconsistently reported among those studies.

Out of the four cross-sectional studies included, three had a low risk of bias [17,27,31], while one study had a medium risk of bias [32]. The observational studies, especially the case–control and cross-sectional studies, had a higher risk of publication bias due to a lack of proper registration procedures. Hence, the overall bias measured in these study designs is higher compared to clinical trials.

### 3.4. Certainty of Evidence (GRADE Analysis) (Table 7)

The certainty of the evidence from randomized control trials for the effect of salivary cortisol on the development of TMD was low (Table 7). The certainty was downgraded because of indirectness and imprecision, as according to the RCTs included, the level of salivary cortisol has minimal or no effect on the development of TMD. Evidence of the effect of salivary cortisol on the development of TMD in the observational studies was also downgraded due to indirectness, impression, and risk of bias. Moreover, for the observational studies all the four criteria for certainty of evidence were downgraded.

Because the certainty of their evidence for the effect of salivary cortisol on TMD development was high quality, randomized control trails and observational studies were included. Therefore, the certainty of evidence for all the included studies was low and IMPORTANT. This result is of IMPORTANCE because salivary cortisol levels are considered as a critical outcome measure in the development of TMDs.

**Table 2:** Characteristics of included studies.

Author, Year, Region	Study Design	Age Range/ Average (yrs)	Sample Size (Test/Control)	Study Population	Key Findings	Conclusion
Rosar et al., 2021, Brazil [30]	Cross-sectional	19–30	43 (28/15)	TMD group Healthy group	Similar salivary cortisol levels found between groups on awakening and after 30 min	Cortisol levels were not associated with the number or duration of bruxism (TMD) episodes
Venkatesh et al., 2021, India [32]	Cross-sectional	18–23	44 (22/22)	Test with TMD Controls without TMD	Salivary cortisol levels showed statistically significant difference between the TMD and control groups	Salivary cortisol can be used as a biological marker of stress in TMD
Goyal et al., 2020, India [28]	RCT	24.05 ± 2.3	60 (20/20/20)	TMDs and positive depression levels TMDs and no depression Healthy control	Statistically significant higher value of salivary cortisol in TMD with depression, as compared to TMD without depression and control	Salivary cortisol could be a promising tool in identifying underlying psychological factors associated with TMDs
D’Avilla, 2019, Brazil [17]	Cross sectional	25.3 ± 5.1	60 (45/15)	Group I: No TMD and clinically normal occlusion Group II: With TMD and malocclusion Group III: TMD and clinically normal occlusion Group IV: No TMD and with malocclusion	Salivary cortisol level was significantly higher in individuals with TMD (G2 and G3), independent of the presence/absence of malocclusion	Quality of life, pain, and emotional stress are associated with and impaired by the TMD condition, regardless of malocclusion presence
Bozovic et al., 2018, Bosnia and Herzegovina [33]	Case–control	19.35	60 (30/30)	TMD group Healthy controls	Levels of salivary cortisol were found to be significantly higher in the study group compared to the control group	Salivary cortisol plays a vital role in TMD development
Chinthakanan et al., 2018, Thailand [34]	Case–control	24	44 (21/23)	TMD group Control group	The salivary cortisol level of the TMD group was significantly greater than that of the control group	Patients with TMD demonstrated autonomic nervous system (ANS) imbalance and increased stress levels
Magri et al., 2018, Brazil [29]	RCT	18–40	64 (41/23)	Laser group (TMD) Placebo group Without treatment group	Women with lower cortisol levels (below 10 ng/mL) were more responsive to active and placebo laser treatment than women with higher cortisol levels (above 10 ng/mL)	Most responsive cluster to active and placebo LLLT was women with low levels of anxiety, salivary levels below 10 ng/mL
Rosar et al., 2017, Brazil [27]	RCT	19–30	43 (28/15)	Sleep bruxism group (Gsb) Control group (Gc)	Salivary cortisol showed a significant decrease between baseline and T1 in test, which was not observed in control	Short-term treatment with interocclusal splints had positive affect on salivary cortisol levels in subjects with sleep bruxism
Poorian et al., 2016, Iran [37]	Case–control	19–40	41 (15/26)	TMD patients Healthy people	Salivary cortisol levels in TMD patients are significantly higher than in healthy people	Increase in salivary cortisol levels increases the probability of suffering from TMD
Tosato et al., 2015, Brazil [31]	Cross-sectional	18–40	49 (26/25)	Women with TMD Healthy women	Moderate to strong correlations were found between salivary cortisol and EMG activities of the women with severe TMD	Increase in cortisol levels corresponded with greater muscle activity and TMD severity
Almeida et al., 2014, Brazil [16]	Case–control	19–32	48 (25/23)	With TMD Without TMD	Results show no difference between groups	No relationship between saliva cortisol, TMD, and depression
Nilsson and Dahlstrom, 2010, Sweden [36]	Case–control	18–24	60 (30/30)	RDC/TMD criteria I RDC/TMD criteria II Control group with no TMD	No statistically significant differences were found between any of the groups	Waking cortisol levels were not associated with symptoms of TMD and were not differentiated between the groups
Quartana et al., 2010, USA [38]	Case–control	29.85	61 (39/22)	TMD patients Healthy controls	Pain index was not associated with cortisol levels	There was no association between markers of pain sensitivity and adrenocortical responses
Jones et al., 1997, Canada [35]	Case–control	27.07	75 (36/39)	TMD group Control group	No significant differences found between TMD and control cortisol levels at baseline, but values were significantly higher in the TMD group at both 30 and 50 min	No relationship was found between psychological factors and hypersecretion of cortisol in TMD group

Temporomandibular joint (TMJ), temporomandibular disorder (TMD), randomized controlled trial (RCT), low-level laser therapy (LLLT), research diagnostic criteria (RDC).

**Table 3:** Salivary parameters of the included studies.

Study (Author, Year)	Saliva Collection	Salivary Cortisol Levels in Tests/Morning/Night	Salivary Cortisol Levels in Controls	Statistical Significance
Rosar et al., 2021 [30]	Stimulated saliva Collection time: immediately after waking up and 30 min after waking up	Upon waking: 0.19 ± 0.21, After 30 min: 0.24 ± 0.28 μg/dL	Upon waking: 0.16 ± 0.13, After 30 min: 0.16 ± 0.09 μg/dL	No *p* > 0.05
Venkatesh et al., 2021 [32]	Stimulated saliva Collection time: 9:30 a.m. to 10:00 a.m.	1.107 ± 0.17	0.696 ± 0.16	Yes *p* < 0.001
Goyal et al., 2020 [28]	Unstimulated saliva Collection time: twice between 7.00 and 8.00 h, and again between 20.00 and 22.00 h	Morning: TMD with depression: 52.45 ± 18.62 TMD without depression: 20.35 ± 10.59 Evening: TMD with depression: 28.13 ± 10.88 TMD without depression: 12.33 ± 6.15	Morning: 12.85 ± 4.28 Evening: 8.51 ± 4.32	Yes *p* = 0.0001
D’Avilla, 2019 [17]	Stimulated whole saliva was collected	G2: 7.45 ± 4.93, G3: 7.87 ± 3.52, G4: 4.35 ± 2.59 μg/dL	3.83 ± 2.72 μg/dL	Yes *p* < 0.05
Bozovic et al., 2018 [33]	Stimulated saliva	2.8 µg/dL	0.6 µg/dL	Yes *p* < 0.001
Chinthakanan et al., 2018 [34]	Unstimulated saliva Collection time: morning, over five minutes	29.78 ± 2.67 ng/ml	22.88 ± 1.38 ng/mL	Yes *p* < 0.05
Magri et al., 2018 [29]	Unstimulated saliva Collection time: between 7 and 10 a.m.	Under 10 ng/mL: 5/7 Above 10 ng/mL: 15/14	Under 10 ng/mL: 6 Above 10 ng/mL: 17	Yes *p* < 0.05
Rosar et al., 2017 [27]	Stimulated saliva Collection time: morning	Baseline: 5.9, T1: 2.6, T2: 2.5	Baseline: 4.9, T1: 4.4, T2: 4.3	Yes *p* < 0.05
Poorian et al., 2016 [37]	Unstimulated saliva Collection time: between 9–11 a.m.	29.0240 ± 5.27835 ng/ml	8.8950 ± 9.58974 ng/mL	Yes *p* = 0.000
Tosato et al., 2015 [31]	Unstimulated saliva Collection time: between 8 and 9 a.m.	Mild: 25.39, moderate: 116.7, severe: 250.1 µg/dL		Yes *p* < 0.05 for moderate and severe
Almeida et al., 2014 [16]	Unstimulated saliva Collection time: between 9:00 and 9:25 a.m.	0.272 µg/dL	0.395 µg/dL	No *p* = 0.121
Nilsson and Dahlstrom, 2010 [36]	Stimulated saliva	10.53 ± 5.05/12.61 ± 8.17 nmol/L	13.68 ± 9.96 nmol/L	No *p* > 0.05
Quartana et al., 2010 [38]	Stimulated saliva Collection time: immediately prior to the start of pain testing, immediately following the pain testing procedures, and 20 min after the pain testing procedures	High PCS: BL: 0.8 Post-pain: 0.85 20 min after pain: 0.9 µg/dL	Low PCS: BL: 0.92 Post-pain: 0.75 20 min after pain: 0.7 µg/ml	No *p* > 0.05
Jones et al., 1997 [35]	Unstimulated saliva Collection time: baseline (time, 0 min), peak secretion (time, 30 min), and after 20 min of rest (time, 50 min)	0 min: 6.41, 30 min: 11.96, 50 min: 10.28	0 min: 5.89, 30 min: 7.63, 50 min: 6.39	Yes *p* ˂ 0.01

Temporomandibular disorder (TMD), randomized controlled trial (RCT), pain catastrophising scale (PCS).

**Table 4:** Risk of bias of randomized controlled trials using Cochrane Risk of Bias Tool II.

Authors/Year	Randomization Process	Deviation from Intended Intervention	Missing Outcome Data	Measurement of the Outcome	Selection of the Reported Results	Overall Bias
Goyal, 2020 [28]	Low	Low	Low	Some concern	Low	Low
Magri, 2017 [29]	Low	Low	Low	Low	Low	Low
Rosar, 2017 [27]	High	High	Low	High	High	High

**Table 5:** Risk of bias of case–control studies using the Newcastle–Ottawa quality assessment scale.

Author, Year	Selection				Comparability	Exposure			
	Is the Case Definition Adequate?	Representativeness of the Cases	Selection of Controls	Definition of Controls	Comparability of Cases and Controls Based on the Design or Analysis	Ascertainment of Exposure	Same Method of Ascertainment for Cases and Controls	Non-Response Rate	Risk of Bias
Almeida et al., 2014 [16]	1	0	1	1	1	0	1	1	Medium (6)
Bozovic et al., 2018	1	1	1	1	1	1	1	1	Low (8)
Chinthakanan et al., 2018 [34]	1	1	1	1	0	0	1	1	Medium (6)
Jones et al., 1997 [35]	1	1	0	1	1	0	1	1	Medium (6)
Nilsson and Dahlstrom, 2010 [36]	1	1	0	1	1	0	1	0	Medium (5)
Poorian et al., 2016 [37]	1	0	0	0	0	0	1	1	High (3)
Quartana et al., 2010 [38]	1	1	1	1	1	1	1	1	Low (8)

**Table 6:** Risk of bias of cross-sectional studies using the Newcastle–Ottawa quality assessment scale.

	Representativeness of the Sample	Sample Size	Non-Respondents	Ascertainment of the Exposure (Risk Factor)	The Subjects in Different Outcome Groups are Comparable, Based on the Study Design or Analysis; Confounding Factors are Controlled	Assessment of the Outcome	Statistical Test	Risk of Bias
D’Avilla, 2019 [17]	1	1	1	2	1	1	1	Low (8)
Rosar et al., 2021 [30]	1	1	1	2	1	1	1	Low (8)
Tosato et al., 2015 [31]	1	1	1	1	1	2	1	Low (8)
Venkatesh et al., 2021 [32]	1	1	0	1	0	1	1	Medium (5)

**Table 7:** GRADE analysis for certainty of evidence.

No. of Studies	Certainty assessment						Effect			Certainty	Importance
	Study Design	Risk of Bias	Inconsistency	Indirectness	Imprecision	Other Considerations	No. of Events	No. of Individuals	Rate (95% CI)		
3	Randomized trials	not serious	serious ^a^	serious ^b^	very serious ^c^	Strong association; all plausible residual confounding would reduce the demonstrated effect	We cannot provide examples extracted from our review since our review was not intentionally limited to a specific prognostic factor. Instead, our goal has been to explore salivary cortisol levels at different times of day that have been investigated to date as potential risks for the persistence of a variety of chronic pain conditions and their associated TMDs. However, this poor representation would happen, for instance, if we were interested in exploring the effects of various levels of salivary cortisol on types of TMD. The studies included were only investigating the prognostic effect of salivary cortisol on TMD at a specific age.			⨁⨁◯◯ Low	IMPORTANT
4	Observational studies (cross-sectional)	serious ^d^	serious ^e^	not serious	serious ^f^	All plausible residual confounding would suggest spurious effect, while no effect was observed	151	196		⨁⨁◯◯ Low	IMPORTANT
7	Observational studies (case–control)	serious ^g^	not serious	serious ^h^	serious ^i^	Publication bias strongly suspected; strong association; all plausible residual confounding would suggest spurious effect, while no effect was observed	When conducting comprehensive systematic reviews of the effects of cortisol levels on TMD incidence among young adults, authors reported that the evidence of increasing salivary cortisol as a prognostic factor for chronic TMD pain has serious limitations. This evidence comes from four studies, and all of them have a moderate risk of bias.			⨁⨁◯◯ Low	IMPORTANT

Explanations: ^a^ Variations in effect estimates across studies, with points of effect on either side of the line of no effect, and confidence intervals showing minimal overlap. ^b^ No precision in the estimation of the effect size within each primary study. ^c^ (1) Sample size justification is not provided and there are fewer than 100 cases reaching the endpoint (for continuous outcomes), and (2) no precision in the estimation of the effect size within each primary study. ^d^ Serious limitations, as most evidence is from studies with a moderate or unclear risk of bias for most bias domains. ^e^ Unexplained heterogeneity or variability in results across studies, with differences in results not clinically meaningful. ^f^ There are few studies and a small number of participants across the studies. ^g^ Serious limitations, as most evidence is from studies with a moderate or unclear risk of bias for most bias domains. ^h^ The study sample, the prognostic factor, and/or the outcome of the primary study do not accurately reflect the review question. ^i^ There are few studies and a small number of participants across the studies.

## 4. Discussion

This systematic review evaluated the relationship between salivary cortisol levels and temporomandibular disorder in young adult patients. The included studies suggest higher salivary cortisol levels in TMD patients than in healthy individuals. Cortisol is a hormone released by the adrenal glands in response to stress. Elevated cortisol levels over an extended period can have negative effects on the musculoskeletal system, including the muscles and joints around the temporomandibular joint (TMJ). Salivary cortisol levels can also serve as a diagnostic marker for evaluating the severity of stress experienced by an individual. Additionally, monitoring cortisol levels over time can provide valuable information about the effectiveness of stress management techniques in reducing TMD symptoms.

Higher cortisol levels were found in TMD patients compared to healthy participants in ten studies included in this systematic review (case–control and cross-sectional) [17,27,28,29,30,31,32,33,34,37]. In contrast, four studies demonstrated no significant association. Two randomized control trials showed reductions in salivary cortisol levels after treatment in patients with TMD compared to those treated with a placebo [28,29]. The possible correlation between TMD and higher salivary cortisol levels could be due to the etiological factors—mainly stress, anxiety, and depression. Recent studies have shown a positive association between stress, anxiety, and depression and the occurrence of TMD [6,39]. These psychological factors were eventually found to be associated with increased salivary cortisol levels.

### 4.1. Association of Cortisol and TMD

For decades, the measure of acute and chronic pain has been demonstrated by patients’ responses to stress meters. Cortisol levels have a vital role in evaluating the level of stress. Cortisol is a glucocorticoid hormone secreted by the adrenal cortex. The activation of gluconeogenesis, the amplification of hepatic protein synthesis, and an increase in the utilization of fat for energy production are among the physiological effects of cortisol [40]. The hypothalamus increases the release of this hormone, known as the stress hormone, when a stressful condition occurs [6]. Stress is believed to not just function as an etiological factor, but also to exacerbate the symptoms of TMD, including pain in the TMJ area [39]. Stress affects the release of cortisol, an anti-inflammatory agent helping to mobilize glucose reserve and modulate inflammation. However, in prolonged or exacerbated stress responses, altered cortisol release leads to inflammation and pain [41]. Stress also activates the HPA axis, resulting in a cascade of responses leading to increased cortisol release from the adrenal cortex. Concerning TMD, patients frequently exhibit hyperactivity of the HPA axis [12]. The hyper-response of the HPA axis has been observed in anxiety disorders and depression. It is also important to note that the mediators for stress response also regulate pain modulation, which is why stress contributes to pain transmission and perception [12].

Various explanations for the association between painful TMD and depression or anxiety are given in the literature. Firstly, these symptoms may trigger muscle hyperactivity, followed by muscle abnormality and pain [42]. They may also induce a joint inflammatory process followed by biomechanical changes, leading to joint pain. Secondly, TMD may be associated with an abnormal trigeminal system process due to imbalances in neurotransmitters like serotonin and catecholamines [40].

### 4.2. Evidence from Randomized Controlled Trials

Three randomized control trials were included in this systematic review. All included studies have reported a positive association between higher cortisol levels and TMD [27,28,29]. Goyal et al. demonstrated a positive association between salivary cortisol levels and TMD in conjunction with depression compared to controls. The authors measured salivary cortisol levels in the morning and evening. In their findings, TMD patients with depression had higher cortisol levels than TMD patients without depression and healthy controls [28]. Student’s *t*-test was performed to measure the difference in cortisol levels between males and females, and it was reported that females with TMD and depression had higher cortisol levels than their male counterparts. The study’s design was a double-blinded control trial where the patients and investigators were unaware of the groups. Hence, the methodological quality of this study is high, and the results are conclusive.

Rosar and colleagues, in their parallel arm randomized trial, concluded that patients with sleep appliances had self-reported TMD with higher cortisol levels in the morning compared to the control. Researchers have also reported that females with TMD and occlusal appliances had higher cortisol levels than males. The authors of this study were unable to calculate awake bruxism. However, the findings of this study suggest higher cortisol levels in self-reported TMD patients compared to controls [27].

Magri et al., in their double-blinded randomized trial, assessed the effect of laser treatment on TMD patients and evaluated the salivary cortisol levels of affected females. The laser treatment was performed over two sittings across eight weeks, and the placebo patients received a light treatment at similar intervals. The salivary cortisol levels were reduced in females receiving laser treatment for TMD compared to controls. The salivary cortisol levels for controls were measured above 10 ng/mL compared to female receiving laser treatment. Findings of all the included RCTs in this review concluded that females with TMD had higher levels of salivary cortisol in the morning compared to controls [29].

### 4.3. Evidence from Case–Control Studies

The six case–control studies included in this review showed mixed results of salivary cortisol and TMD levels [33,34,35,36,37,38]. Four studies measured a positive association [33,34,37], while three studies indicated no significant correlation between the salivary marker and TMD [16,36,38]. Jasim et al. compared females with chronic and acute TMD and suggested no statistical significance in the levels of salivary cortisol among the group, even though perceived stress and depression were marked in both groups [43]. Similar findings were reported in the study by Almeida and colleagues, which compared individuals with and without TMD, with 64% of patients with TMD reporting having depression. Saliva cortisol levels were 0.272 g/dL in the group with TMD and 0.395 g/dL in the group without it, with no statistical significance [16]. In line with these results, studies by Nilsoson and Dahlstrom and Quartana reported no significant difference in salivary cortisol levels in patients with and without TMD. However, both studies showed a higher level of salivary cortisol in female TMD patients than their male counterparts [36,38].

On the other hand, Chinthakanan et al. reported a significant increase in cortisol levels in TMD patients compared to a control group; they concluded that TMD patients demonstrated an autonomic nervous system (ANS) imbalance and elevated stress levels [34]. Similarly, the study by Bozovic et al. on students with and without TMD reported a significantly higher level of salivary cortisol in TMD students with depression and stress [33]. The overall results of all the included studies report that females with TMD, depression, and stress have higher levels of salivary cortisol.

### 4.4. Evidence from Cross-Sectional Studies

Among the included cross-sectional studies (*n* = 43) [30] in this review, one half found no association between TMD and salivary cortisol, while the other showed a positive correlation [17,31,32]. The research by Tosato et al. correlated the cortisol concentration in saliva with the bioelectric activity of masticatory muscles [31]. They showed elevated levels of glucocorticosteroids with increased muscle tone and pronounced severity of TMD symptoms. Their work agrees with the hypothesis that increased muscle activity corresponds with hyperactivity of the HPA axis [7].

D’Avilla et al. indicated in their study that the concurrence of malocclusion and TMD has a significant negative influence on masticatory capacity, while salivary cortisol levels are higher in TMD patients regardless of the presence or absence of malocclusion. In contrast, the study by Rosar et al. reported no statistical significance of the level of salivary cortisol between the groups; moreover, the cortisol level was not increased in patients with psychological impairments. In conjunction with the findings of the case–control studies and RCTs included in this review, the cross-sectional studies also reported higher salivary cortisol in young females with TMD, stress, and depression than their male counterparts [17].

### 4.5. Evidence from Systematic Reviews

Two systematic reviews on a similar topic were found in the literature. Lu et al. performed a meta-analysis of thirteen studies, including case–control studies, and stated the occurrence of increased cortisol levels in TMD patients. However, these results were inconclusive due to high heterogeneity among the included studies [21]. Their review included studies involving children and adult patients, while this review focused mainly on adults. Studies have suggested that adults aged 18–40 have higher stress levels than children and the elderly. Treatment of TMD and its associated psychological factors should be prioritized in this age group more than children and the elderly. Fritzen and colleagues performed the other systematic review; they checked salivary cortisol concentration in adults and children with bruxism and incorporated six studies into their data [20]. They concluded a positive correlation between bruxism and higher cortisol levels in saliva in patients with higher stress levels. Similar findings are reported in this review, but to strengthen our results, we incorporated all the clinical study designs and restricted the age group from 18–40 years.

To the best of our knowledge, this is the first review that focuses on the relationship between salivary cortisol levels and TMD in young adult patients, along with the patients’ salivary cortisol concentrations. In this review, the authors have included every study’s design to strengthen the findings. However, there are a few limitations to this systematic review. Firstly, heterogeneity was observed in the included studies; hence, a meta-analysis could not be performed. Secondly, gender dimorphism was not evaluated in the studies, as a few included only female patients [29], while in other studies, the female–male ratio was unequal [16]. Even though Quartana et al. stated in their results that there is a significant correlation between cortisol levels and TMD, the data they provided regarding salivary cortisol levels were unclear [38].

Furthermore, there were disparities in the timing of saliva collection among the studies; seven studies collected salivary samples in the morning [27,29,30,31,32,34,37,38,44], while one study collected it both in the morning and evening [28]. One study sampled saliva before and after the stress test [38]. Finally, due to the small sample size of the included studies, a conclusion cannot be drawn. Hence, more longitudinal studies with robust methodological approaches should be designed to support the findings of this review.

## 5. Conclusions

This systematic review demonstrates a relationship between salivary cortisol levels and TMD. The salivary cortisol levels were higher in TMD adults than healthy controls, which could be linked to probable exposure to stress, depression, and anxiety. Moreover, females suffering from TMD in conjunction with depression and stress had higher salivary cortisol levels. This is suggestive of the need for psychological and clinical treatment in patients with TMD. Considering the high heterogeneity and inconsistency of results in the included studies, higher-quality studies, with bigger sample sizes and follow-ups, are required.

## Figures and Tables

**Table 1:** Search string for various databases.

PubMed	(hydrocortisone) “[MeSH Terms] OR “hydrocortisone”[All Fields] OR “cortisol”[All Fields]) AND (“Temporomandibular disorder”[MeSH Terms] OR “TMD”[All Fields]) OR (“temporomandibular disfunction”[MeSH Terms] OR (“Facial muscle pain”[All Fields] AND young Adults [All Fields].
Scopus	(TITLE-ABS-KEY (“craniomandibular disorder*” OR “temporomandibular joint disorder*” OR “temporomandibular disorder*” OR tmjd OR tmd OR “tmj disorder*” OR ((facial OR jaw OR orofacial OR craniofacial OR trigem*) AND pain))) AND (TITLE-ABS-KEY (pcs OR “Salivary cortisol” OR Hydrocortisone* OR cortisol AND (Young adults))))
Web of science	cortisol* OR hydrocortisone* AND Temporomandibular disorder* OR TMD* AND Young adults.
Google scholar	(cortisol OR Salivary cortisol AND Temporomandibular disorder OR TMD AND Young Adults).

## Data Availability

The data that support the findings of this study are available on request from the corresponding author.
